# Effects of a Specialized Oral Nutritional Supplement with Dietary Counseling on Nutritional Outcomes in Community-Dwelling Older Adults at Risk of Malnutrition: A Randomized Controlled Trial

**DOI:** 10.3390/geriatrics9040104

**Published:** 2024-08-17

**Authors:** Weerasak Muangpaisan, Sanit Wichansawakun, Dieu Thi Thu Huynh, Somboon Intalapaporn, Chalobol Chalermsri, Ornicha Thititagul, Kanokkarn Chupisanyarote, Mallika Chuansangeam, Arunee Laiteerapong, Menaka Yalawar, Chengrong Huang, Siew Ling Tey, Zhongyuan Liu

**Affiliations:** 1 Faculty of Medicine Siriraj Hospital, Mahidol University, Bangkok 10700, Thailand; drweerasak@gmail.com (W.M.);; 2 Faculty of Medicine, Thammasat University, Pathum Thani 12120, Thailand; 3 Abbott Nutrition Research and Development, Asia-Pacific Center, Singapore 138668, Singapore; 4 Thammasat University Hospital, Thammasat University, Pathum Thani 12120, Thailand; 5 Abbott Nutrition Research and Development, Bangkok 10120, Thailand; 6 Biostatistics and Statistical Programming, Cognizant Technologies Solution Pvt. Ltd., Bangalore 560092, India; 7 Abbott Nutrition Research and Development, Shanghai 200233, China

**Keywords:** older adults, malnutrition, oral nutritional supplement, beta-hydroxy-beta-methylbutyrate (HMB), community, nutritional status, dietary counseling

## Abstract

This study investigated the effects of oral nutritional supplements (ONSs) along with dietary counseling (DC) in community-dwelling older adults at risk of malnutrition. In this randomized controlled trial, 196 older adults who were at risk of malnutrition, as identified by the Malnutrition Universal Screening Tool (MUST) were randomly assigned to receive ONSs twice daily with DC (intervention) or DC-only (control) for 60 days. Primary outcome was change in body weight from baseline to day 60. Nutritional status, energy, and macronutrient intakes were measured. A significant larger weight gain was observed in the intervention compared to the control from baseline to day 60 (1.50 ± 0.22 kg, *p* < 0.0001). The intervention group also showed a significantly greater increase in weight at day 30 (*p* < 0.0001). Intakes of energy and macronutrients were significantly higher in the intervention group compared to the control group at both days 30 and 60 (all *p* < 0.0001). The odds of achieving better nutritional status were significantly higher in the intervention group than in the control group (OR:3.9, 95% CI: 1.9, 8.2, *p* = 0.0001). ONS supplementation combined with DC significantly improved body weight and nutritional outcomes in community-dwelling older adults at risk of malnutrition.

## 1. Introduction

The global population of older adults aged 65 years and older is expected to rise from 761 million in 2021 to 1.6 billion by 2050, posing significant healthcare and socioeconomic challenges [1]. Several countries in Southeast Asia (SEA) are projected to experience a higher rate of increase in the proportion of their aging population when compared to Western countries [1]. In 2023, Thailand was classified as a ‘complete aged society’ [2], with the second highest aging population (≥65 years) in SEA (14% of its total population) after Singapore (17%) and followed by Vietnam (10%), Myanmar, Malaysia, and Indonesia (7% each) [3]. By 2042, the total Thai population is expected to decrease to 60 million. On the other hand, older persons (aged 60 years or older) in Thailand is projected to increase to 19 million, accounting for ~31.4% in 2042 [4].

Age-related physiological and psychological changes make older adults highly susceptible to malnutrition [5,6,7,8]. Besides age, the risk of malnutrition is influenced by other factors including health status, and healthcare settings [7,9]. Systematic reviews and meta-analyses have determined the prevalence of malnutrition in older adults and reported higher estimates in hospital, followed by residential care and community settings, both globally and in Asia [10,11]. Cereda et al. conducted a meta-analysis of 240 studies and reported a worldwide pooled prevalence of malnutrition (Mini Nutritional Assessment [MNA^®^] score < 17) of 3.1% and 22.0% (4.8% and 26.2% in Asia) in the community and hospital settings, respectively. The corresponding estimates for the risk of malnutrition (MNA^®^ score 17–23.5) were 26.5% and 45.6% worldwide, and 29.8% and 48% in Asia [10]. Another meta-analysis of 196 studies by Leij-Halfwerk et al. reported a 8.5% risk of malnutrition in community-dwelling older adults compared to 17.5% and 28.0% in residential care and hospital settings [11]. Crichton et al. analyzed 111 studies and found that the pooled prevalence of protein–energy malnutrition among community-dwelling older adults was 24.6% in SEA [12]. 

The risk of malnutrition in older adults is high in Thailand, similar to the trends reported worldwide and in Asia. A meta-analysis by Chuansangeam et al. analyzed 71 studies involving older adults in Thailand and reported a pooled prevalence of malnutrition of 6.1%, while the risk of malnutrition was 42.6% [13]. A cross-sectional study conducted in 330 community-dwelling older adults living in Chiang Mai, Thailand, reported that 54.8% were at nutritional risk. Factors influencing malnutrition risk included old age, low income, living alone, moderate to severe pain, dyslipidemia, osteoarthritis, and one or more falls in the previous year [14]. The high malnutrition risk among community-dwelling older adults in Thailand is concerning and demands immediate attention. 

Malnutrition among the older adults can lead to impaired immune status, muscle wastage, reduced cognitive function, delayed recovery from acute illness, prolonged hospital stays, and increased mortality [8,15,16]. Malnutrition in community-dwelling older adults has been associated with low muscle mass and sarcopenia, high rate of disability, poor quality of life (QoL), and functional impairment [17,18,19,20]. The economic burden of malnutrition is also substantial in both community-dwelling and hospitalized older adults [21,22]. Strategies prioritizing early diagnosis and prevention or early management of malnutrition could help mitigate the associated complications and healthcare costs in Thailand’s aging population. The Global Leadership Initiative on Malnutrition 2019 consensus recommends the use of validated screening tools such as the Malnutrition Universal Screening Tool (MUST) for assessment of nutritional risk [23]. Furthermore, strategies such as nursing interventions, education, food modifications, dietary counseling (DC), and oral nutritional supplements (ONSs) are recommended to address malnutrition before the use of intensive treatments, such as enteral or parenteral nutrition [16,24]. 

ONSs are dietary supplements typically containing both macro- and micronutrients. ONSs provide energy and are commonly used to increase energy and nutrient intakes when the regular food intake is inadequate [25]. Beta-hydroxy-beta-methylbutyrate (HMB), a metabolite of amino acid leucine, present in some ONS formulations, has been shown to support muscle health [26,27]. Evidence suggests that use of ONSs can improve weight gain and nutritional status in hospital and post-hospital discharged patients [28]. 

Optimizing the use of ONS formulations in rapidly aging societies like Thailand could be an effective preemptive approach to prevent or tackle malnutrition [29]. Currently, most studies with ONSs in Thailand are conducted in hospitalized adult patients [30], or patients with underlying conditions like kidney failure [31,32]. To the best of our knowledge, no clinical trial to date has been conducted in Thailand to evaluate the effects of ONSs among community-dwelling older adults at risk of malnutrition. Considering that a high proportion of older adults at risk of malnutrition reside in communities in Thailand (~55%) [14], there is a clear pressing need for well-designed studies to determine the health and nutritional benefits of ONSs in this population cohort. The objective of this RCT is to investigate the effects of ONSs along with DC (intervention group) compared with DC alone (control group) on body weight and nutritional outcomes in community-dwelling older adults aged ≥60 years at risk of malnutrition in Thailand. 

## 2. Materials and Methods

### 2.1. Study Design

This was a prospective, multicenter, randomized controlled, open-label, parallel-design study conducted in Thailand. The study team was not blinded given the open-label design. Study participants were recruited from two study centers, including Thammasat University and Siriraj Hospital in Thailand. Eligible study participants (n = 196) were enrolled and randomly allocated to one of the two groups: (1) 2 servings per day of ONSs plus DC (intervention group), or (2) DC alone (control group) for a duration of 60 days. Randomization was stratified by MUST malnutrition status (medium or high risk) and gender. The randomization schedules were computer-generated using a dynamic minimization algorithm. The research was conducted according to the Declaration of Helsinki. All participants voluntarily provided written informed consent before study participation. The study was approved by the Institutional Review Board at the participating centers in Thailand. The study was registered with ClinicalTrials.gov, NCT05682781.

### 2.2. Study Population

Participants were required to meet the following inclusion criteria: individuals aged 60 years or above who were at medium or high risk of malnutrition as defined by the MUST, community-dwellers, defined as individuals that did not stay in a residential intermediate or long-term care service institution, and community ambulant with or without aid. Participants did not have any chronic disease(s) or had stable chronic disease(s), which was defined as any long-term medical condition on regular medications that had not changed 90 days prior to the baseline visit. Participants were able to consume food and beverages orally, communicate, and follow instructions. Participants were willing to refrain from taking non-study ONSs, follow study procedures and record data in a diary, and complete any forms or assessments needed throughout the study. Individuals were excluded if they had any of the following conditions: patients with type 1 or type 2 diabetes, active infectious disease (e.g., tuberculosis, Hepatitis B or C, HIV infection), severe gastrointestinal disorders (e.g., celiac disease, short bowel syndrome, pancreatic insufficiency, cystic fibrosis), advanced renal disease or end-stage organ or pre-terminal diseases, acute myocardial infarction within the last 30 days, active malignancy within the last five years or currently undergoing treatment for malignancy, eating disorders, dementia or delirium, history of significant neurological or psychiatric disorder, alcoholism, substance abuse, allergy or intolerance to milk products, continuous ONS usage for 30 days prior to the baseline visit, taking any herbals, dietary supplements, medications during the past 30 days prior to the baseline visit that could profoundly affect body weight or appetite, clinically significant ascites, pleural effusion, edema, or dehydration, etc.

### 2.3. Study Treatment

Participants in the control group received only DC, while participants in the intervention group received DC plus two servings per day of ONSs for 60 days. The ONS (Ensure, Abbott Nutrition, Zwolle, the Netherlands) used in the study was a ready-to-drink liquid. It provides complete and balanced nutrition, and contains 270 kcal, 11 g protein, 9 g fat, 34.5 g carbohydrate, and 0.74 g calcium HMB per 220 mL serving (Supplementary Materials Table S1). The study ONS was used as a supplement, in addition to participant’s usual intake. DC was given by the study dietitian, or a trained researcher. Dietary counseling was conducted at baseline and day 30. The content of DC was developed based on the European Society for Clinical Nutrition and Metabolism (ESPEN) practical guidelines for clinical nutrition and hydration in geriatrics [16]. As the guiding value, the target energy intake was 30 kcal per kg of actual body weight per day, and protein was at least 1 g protein per kg of actual body weight per day. The participant’s energy and nutrient requirements were estimated and individually adjusted based on their nutritional status, degree of physical activity, disease status, tolerance, etc. Dietary counseling was conducted based on participant’s nutritional requirements and tailored according to their underlying conditions and food allergy (if any), food preference, and cultural practice. Participants in the control group were counseled on food selection and meal planning, including snacks in addition to main meals, with the aim of achieving the energy and nutrient requirements. Participants in the intervention group was counseled to consume 2 servings of study ONS as part of their diet to help achieve their energy and nutrient requirements. Consuming non-study ONSs including protein powder supplements was not permitted during the study.

### 2.4. Study Endpoints and Assessments

Data were collected from each participant during the three study visits: baseline, day 30, and day 60. The primary endpoint was the change in body weight from baseline (day 0) to day 60. Secondary outcomes included assessment of malnutrition risk using MUST, change in body weight from baseline to day 30, and changes in body mass index (BMI) from baseline to days 30 and 60. Additionally, energy and macronutrient intakes were collected at baseline, and days 30 and 60. 

Dietary assessment was conducted using a single 24 h dietary recall at each visit to determine participant’s energy and dietary intakes. The dietitian or trained researcher conducted the recall at each visit and asked the participants about the food and drink consumed on the previous day. Nutrient analysis was performed using the dietary analysis program INMUCAL (version 4.0), which is a software developed by the Mahidol University Institute of Nutrition. Malnutrition risk was determined using MUST, which is based on the scores related to BMI, unintentional weight loss, and acute disease and nutritional intake [33]. Participants were categorized into one of three nutritional status categories: low risk/score = 0, medium risk/score = 1, and high risk/score ≥ 2; a higher score indicated increased risk of malnutrition [33]. 

Sociodemographic and characteristics of participants such as height, age, gender, education, main work status, and living area were collected during the baseline visit. The Charlson Comorbidity Index (CCI) was collected at baseline to measure the level of comorbidity [34].

Compliance with product intake was calculated over 60 days using participant’s intake record and return of unused products. Percentage of compliance was calculated using the following formula: number of bottles consumed divided by number of bottles instructed to consume, multiplied by 100. 

### 2.5. Statistical Analysis

The SHIELD study [29] showed a significant mean weight change difference of 0.84 kg (standard deviation [SD], 1.8 kg) compared with the placebo group after 60 days of ONS treatment. This difference was clinically significant with respect to improvement in health and nutritional outcomes. To detect an effect size of 0.47 [29] with 80% power using a two-group *t*-test with 0.05 two-sided significance level (SAS^®^ version 9.4), we calculated a sample size of 73 participants per study group. Considering an attrition rate of approximately 25%, we aimed to enroll and randomize 98 participants per group (196 total participants).

Continuous variables were analyzed using parametric analyses (such as analysis of variance (ANOVA), analysis of covariance (ANCOVA), repeated measures ANCOVA, and paired *t*-test). The residuals from the parametric analysis were utilized to check for deviation from normality by a combination of methods (stem-and-leaf plot of residuals, normality plot, Shapiro–Wilk test). If the distribution of the variable deviated significantly from normality, suitable nonparametric analysis (such as Wilcoxon rank sum test) was used to analyze the variable. Categorical variables were analyzed using test of association, i.e., chi square test or Fisher’s exact was used for the analysis.

The primary variable, change in body weight from baseline to 60 days, was analyzed using ANCOVA with the main effects for study center, study group, and gender; interactions for study group*gender, study group*baseline MUST risk; and baseline weight and baseline MUST risk as covariates. 

MUST risk at 30 and 60 days were analyzed using Fisher’s exact test (study group) by visit and confirmatory analysis was performed using repeated generalized estimating equations (GEE) model (study center, visit, study group, gender, study group*visit, study group*gender). Energy intake, carbohydrate, fat, and protein at 30 and 60 days were analyzed using repeated measures ANCOVA (study center, visit, study group, gender, baseline MUST risk, study group*visit, study group*gender, study group*baseline MUST risk) using baseline value as covariate. Change in body weight from baseline to 30 days, and percent change in body weight and change in BMI from baseline to 30 day and 60 days were analyzed using ANCOVA with factors for study center, study group, gender, baseline MUST risk, study group*gender, study group*baseline MUST risk, and baseline weight as a covariate. Additionally, change in body weight from baseline to 30 days and 60 days within each study group was analyzed using paired *t*-test. 

All participants who received at least one study feeding (for intervention group only) and all enrolled participants in the control group were included in the intent-to-treat (ITT) analysis. All the analyses were conducted using the ITT dataset. All analyses were performed using SAS^®^ Version 9.4. *p* < 0.05 was considered statistically significant.

## 3. Results

### 3.1. Study Population and Baseline Characteristics

**Figure 1:**
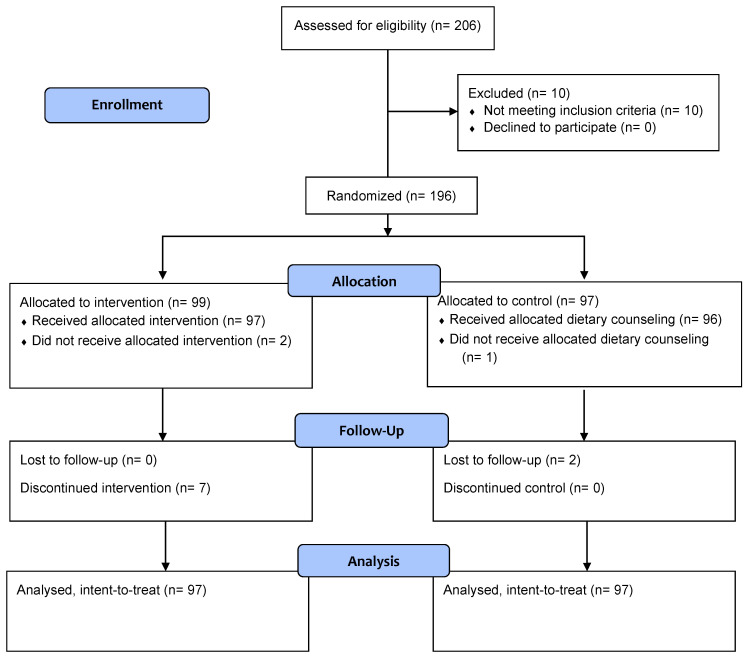
CONSORT study flow diagram.

The study was conducted between March 2023 and January 2024. A total of 196 participants were enrolled and randomized either to the intervention group (n = 99) or the control group (n = 97) (Figure 1). Two participants from the intervention group were randomized but later were found out to be non-eligible for the study. They were excluded from the study and did not receive the study ONS. The rest of 194 participants (97 per group) were included in the intent-to-treat (ITT) analysis. There were no significant differences in baseline socio-demographic characteristics between the two groups (Table 1). Mean body weight was similar between the study groups at baseline (mean ± SE: 42.96 ± 0.37 kg). 

### 3.2. Change in Body Weight and Percentage of Change in Body Weight

**Figure 2:**
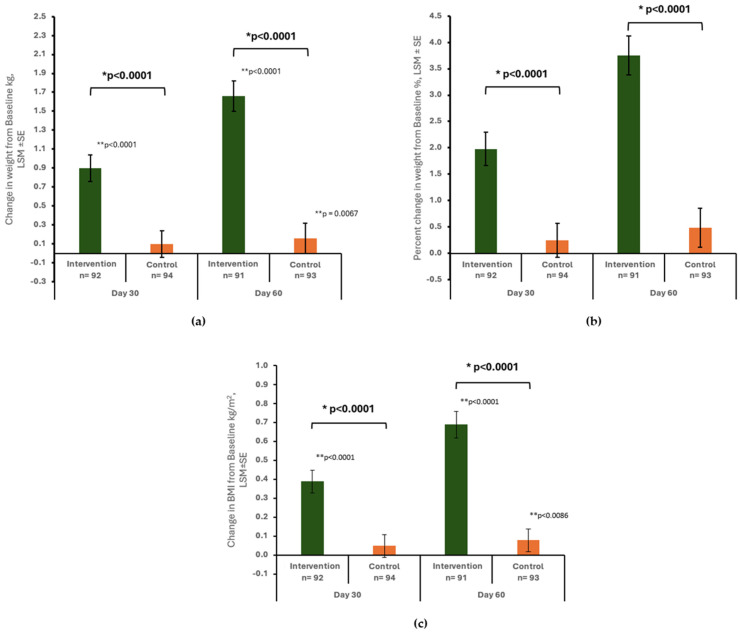
Change in body weight (kg) (**a**), percent change in body weight (**b**), change in BMI (kg/m^2^) (**c**) from baseline to day 30 and day 60 by study group. Data are presented as least squares mean ± standard error (LSM ± SE) unless otherwise specified. * *p*-value from ANCOVA with factors for study center, study group, gender, baseline MUST risk, study group*gender, study group*baseline MUST risk, and baseline value as covariate; ** *p*-value from paired *t*-test for change in body weight (kg) and change in BMI (kg/m^2^) within group.

The change in body weight was significantly greater in the intervention group compared to the control group at day 30 (Figure 2a). Specifically, the gain in weight in the intervention group was nine times the weight gain in the control group (0.90 ± 0.14 kg vs. 0.10 ± 0.14 kg, between-group difference: 0.80 ± 0.19 kg, *p* < 0.0001). This difference became even more pronounced at day 60, with the intervention group achieving ten times as much weight gain as the control group (1.66 ± 0.16 vs. 0.16 ± 0.16 kg, between-group difference: 1.50 ± 0.22, *p* < 0.0001). Additionally, the intervention group demonstrated significant improvement in body weight at days 30 and 60 compared to the baseline (both *p* values < 0.0001). In contrast, significant improvement in body weight was only observed at day 60 in the control group (*p* = 0.0067).

At day 30, the percentage of weight change was significantly greater in the intervention group compared to the control group (1.98 ± 0.32% vs. 0.25 ± 0.32%, between-group difference: 1.73 ± 0.43%, *p* < 0.0001). By day 60, the mean percent of weight change in the intervention and control groups was 3.75 ± 0.37% and 0.48 ± 0.37%, respectively, with a significant difference between groups (3.27 ± 0.50%, *p* < 0.0001) (Figure 2b).

### 3.3. Change in BMI

A significantly larger increase in BMI was found in the intervention group compared to the control group at day 30 (0.39 ± 0.06 kg/m^2^ vs. 0.05 ± 0.06 kg/m^2^, between-group difference: 0.34 ± 0.08, *p* < 0.0001) and day 60 (0.69 ± 0.07 kg/m^2^ vs. 0.08 ± 0.06 kg/m^2^, between-group difference: 0.62 ± 0.09, *p* < 0.0001) (Figure 2c). Within the intervention group, a significant increase in BMI was observed from baseline to days 30 and 60 (both *p* < 0.0001). In contrast, the control group experienced a significant increase in BMI only at day 60 (*p* = 0.0086).

### 3.4. Nutritional Status

**Figure 3:**
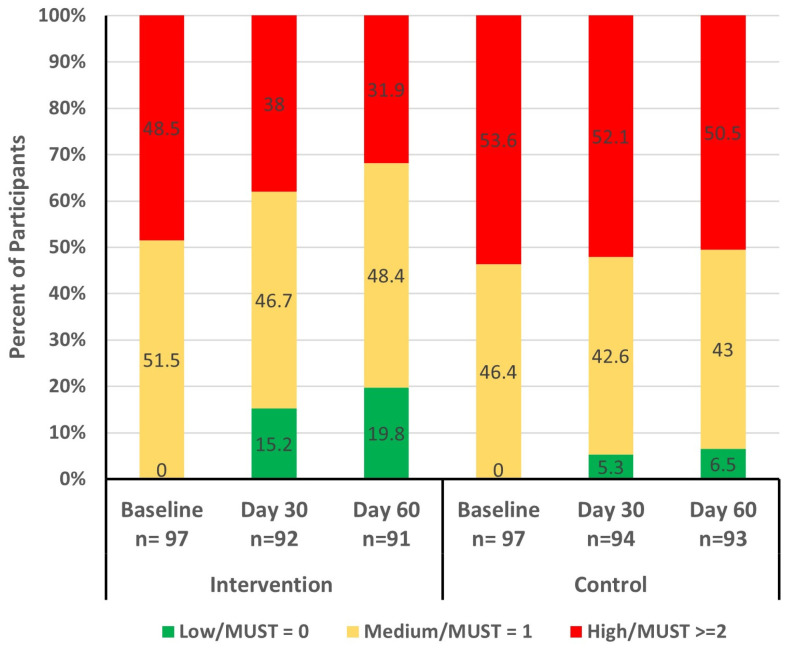
MUST risk (low, medium, and high) at baseline, day 30, and day 60 by study group. MUST: Malnutrition Universal Screening Tool.

At baseline, there were no significant differences between the study groups for nutritional status as determined by the MUST. At day 30, a higher proportion of participants in the intervention group (15.2%) were classified as low risk (MUST score = 0), indicating significantly better nutritional status compared to those in the control group (5.3% with low risk of MUST score = 0; *p* = 0.0366). By the end of the study (day 60), more participants in the intervention group improved nutritional status compared to those in the control group (Figure 3). The odds of achieving better nutritional status (lower MUST score) in the intervention group was significantly higher than the control group during the study period (odds ratio [OR] = 3.9; 95% confidence interval [CI]: 1.9, 8.2; *p* = 0.0001) and at days 30 and 60 (OR = 3.5; 95% CI: 1.4, 8.7; *p* = 0.0053 and OR = 4.4; 95% CI: 1.8, 10.6; *p* = 0.0006, respectively). 

### 3.5. Dietary Intake

As shown in Table 2, the energy intake was significantly higher in the intervention group at days 30 and 60 compared to the control group (*p* < 0.0001 at both timepoints). Similarly, protein, carbohydrate, and fat intakes were significantly higher in the intervention group at days 30 and 60 compared to the control group (all *p* < 0.0001). 

### 3.6. Compliance

The average compliance with ONSs over 60 days was high (92.8%) in the intervention group. Additionally, 94.8% (92/97) of participants from the intervention group and 96.9% (94/97) from the control group completed two dietary counseling sessions at baseline and day 30.

### 3.7. Safety

A total of 29 treatment-emergent adverse events (AEs) from 16 unique preferred terms (PTs) across 8 system organ classes (SOCs) were reported. AEs were reported in 16 (16.5%) and 11 (11.3%) of participants in the intervention and control groups, respectively. ONS-related AEs, mainly those from the SOC gastrointestinal disorders, including abdominal distension (3.1%), diarrhea (3.1%), and gastrointestinal motility disorder (3.1%), were reported in 9.3% of participants and symptoms were of mild or moderate severity. These are common events in this population. Serious AE (SAE) of PT transient ischemic attack was reported in one participant in the control group and none in the intervention group. Overall, there were no statistically significant or clinically relevant trends for any specific PTs reported during this study, and no safety concerns associated with the consumption of the study product.

## 4. Discussion

To the best of our knowledge, this is the first randomized controlled trial (RCT) in Thailand that compares the effects of an ONS containing HMB plus DC with DC alone in community-dwelling older adults at risk of malnutrition. Results show that the intervention using ONSs and DC significantly improved body weight, nutritional status, and energy and macronutrient intakes compared to DC alone. Additionally, a high rate of compliance with the ONS (over 90%) was observed. These findings potentially offer a viable and preemptive approach to prevent nutritional deficiencies and improve nutritional outcomes. Moreover, this study focuses on early nutrition intervention. Approximately 30% of patients are already malnourished upon hospital admission [35]. Tackling malnutrition early in the community settings may help prevent hospital admission and other adverse consequences of malnutrition [36]. Hence, the present study indicates that ONSs in conjunction with DC is an effective strategy to help prevent or reduce the risk of adverse health outcomes, especially those leading to hospitalization, in older adults at risk of malnutrition.

Weight gain serves as an important indicator of improvement in nutritional status for older individuals who are malnourished or at risk of malnutrition [16]. A systematic review and meta-analysis by Baldwin et al. including 931 adults with malnutrition from 14 studies reported a significantly higher weight change up to 3 months in the groups receiving ONSs plus DC compared to DC alone with a mean difference of 1.15 kg (95% CI: 0.42, 1.87) [37]. The SHIELD study, a RCT involving 811 community-dwelling older adults at risk of malnutrition, showed a significant greater increase in weight at 3 months (least squares mean difference ± standard error: 1.07 ± 0.11) in the intervention group receiving ONSs containing HMB with DC, compared to placebo with DC [29]. In line with previous research, the present study shows that including ONSs along with DC is superior to DC alone using a food-based approach in improving nutritional outcomes, including body weight and BMI, for older individuals at risk of malnutrition. Notably, the control group receiving DC alone experienced a small significant increase in body weight only at day 60 (mean change from baseline to day 60: 0.16 kg), compared to the ONSs along with DC, which showed a significant increase in body weight as early as day 30 (mean change at day 30: 0.90 kg; day 60: 1.66 kg). The Global Leadership Initiative on Malnutrition (GLIM) considers weight loss (>5% within past 6 months, or >10% beyond 6 months) as a phenotypic criterion for the diagnosis of malnutrition due to increased risk of negative health consequences [23]. In this study, the intervention group achieved a 3.75% weight gain within 2 months, compared to 0.48% in the control group. When taken together, the use of ONSs along with DC is highly effective in promoting faster recovery of nutritional outcomes in individuals with malnutrition or at risk of malnutrition when compared to DC alone.

Optimizing nutritional screening and assessment and administering individually tailored nutritional preventive or treatment strategies is one of the key principles for managing malnutrition [7,16,38]. Assessing and improving the nutritional status of patients with malnutrition is clinically important, as it helps reduce frailty, enhance physical performance [39], decrease disease-related complications [40], and improve survival [41]. In our study, nutrition screening was performed using the MUST tool. MUST is a simple and reliable nutrition screening tool that can be utilized in the community setting [42]. In the present study, the odds of having better nutritional status were significantly higher in the intervention group compared to the control over the study period (OR = 3.9; 95% CI 1.9, 8.2; *p* = 0.0001), and at day 30 and 60 (OR = 3.5 *p* = 0.0053; OR = 4.4 *p* = 0.0006, respectively). The SHIELD study demonstrated a similar improvement in nutritional status with ONSs containing HMB among community-dwelling older adults from baseline to day 180 (OR = 2.68; 95% CI 1.97, 3.65; *p* < 0.001 vs. placebo), and at days 30, 90, and 180 (OR = 2.29, 3.35, and 2.50, respectively; all *p* < 0.001) [29]. In a separate study involving ONS administration in hospitalized older population with malnutrition, the ONSs containing HMB led to significantly higher odds of better nutritional status, as measured by the subjective global assessment, compared with placebo at day 90 (OR = 2.04, 95% CI 1.28, 3.25; *p* < 0.009) [43]. Peng et al. found a borderline improvement in nutritional status as assessed by MNA-Short Form between the ONS and control groups (*p* = 0.064) [44]. This difference in results could be attributed to various factors, including the different study populations. The study by Peng et al. was conducted among pre-frail older adults, whereas the present study was conducted among older adults at risk of malnutrition. Overall, existing studies indicate that ONSs containing HMB significantly improve nutritional status in older adults at risk of malnutrition, regardless of healthcare settings and the nutritional screening and assessment tool used.

Older adults with malnutrition or at risk of malnutrition often require a high intake of energy and nutrients to achieve improvement in nutritional status [16]. ONS formulations, being energy- and nutrient-dense, are frequently used to improve nutritional status, particularly in cases of malnutrition among older adults [16,24]. The integrated care for older people published by the World Health Organization (WHO) recommends oral supplemental nutrition with dietary advice for older people affected by undernutrition [24]. Similarly, the ESPEN guidelines recommend older persons with malnutrition or at risk of malnutrition with chronic conditions shall be offered ONSs when dietary counseling and food fortification are not sufficient to increase dietary intake and reach nutritional goals [16]. Lately, the expert consensus from the Asian Working Group for Sarcopenia (AWGS) recommends that for older adults who are candidates for supplementation, ONSs containing HMB may be considered [45]. The ONS used in our study is energy-dense and fortified with proteins, HMB, and a good amount of macronutrients and micronutrients. The significant improvements in body weight, BMI, and nutritional status are attributed to the significant increase in energy, carbohydrate, protein, and fat intakes reported with the study ONS. Our findings are consistent with those reported by Chew et al., where a significant increase in energy, protein, fat, and carbohydrate intakes with ONSs led to significant improvement in nutritional outcomes in community-dwelling older adults at risk of malnutrition [29]. The similarity in results could be attributed to the comparable macronutrient and micronutrient composition and serving sizes across both studies. 

Good compliance is essential for maximizing the clinical benefits with ONSs, but it can be challenging to achieve in older adults due to contextual, personal, and product-related factors [46]. A systematic review reveals a significant negative correlation between adherence to ONSs and mean patient age, suggesting poorer adherence in older patients [47]. Studies across various settings and durations have consistently demonstrated that adherence to ONSs positively correlates with improved nutritional status, including improved body weight and reduced risk of malnutrition. A recent systematic review and meta-analysis found that high ONS compliance (≥80%) substantially reduced the incidence of complications such as infections, pressures ulcers, and wound healing among community-dwelling adults with a mean age of 67 years [40]. High compliance (83.5%) with ONSs has also shown to improve QoL, lower the proportion of patients experiencing severe functional impairment [48,49], and reduce the risk of hospitalizations in community-dwelling older adults [50]. A high compliance rate (92.8%) was reported with our study ONS, which may have contributed to the significant improvement in nutritional status. Good palatability and convenience of ONS administration may have resulted in the high compliance rates noted in this study. The high compliance observed in the current study indicates that ONS intervention is both feasible and effective in the community setting for improving nutritional status in older individuals with or at risk of malnutrition.

The study has several strengths and limitations. To reduce the assessment bias in this open-label study, we used objective measurements such as body weight and BMI. Our population choice of community-dwelling older adults reflects a typical real-world scenario where most older adults at malnutrition risk reside in community settings. The high compliance with ONSs achieved in our study portrays the feasibility of incorporating ONSs into clinical practice for the geriatric population who are at risk of malnutrition. Study limitations include the short duration of the study (2 months) compared to most published studies with ONSs that are of ≥3 months duration. However, within one month of supplementation, the results confirmed the beneficial effects of ONSs in improving nutritional status. The utilization of a single 24 h recall for dietary assessment may not fully capture the variability in usual dietary intake due to daily fluctuations, which represents another limitation of our study. Although the three-day food record is a more comprehensive measurement, it also increases the burden of study participants as they need to capture food intakes for three days each at the beginning, middle, and at the end of the study (a total of nine days for the whole study). The 24 h recall is sufficient and adequate for this study, because the main purpose of collecting dietary recall is to estimate the mean intake for each study group. The objective measurement, i.e., change in body weight, is the primary outcome of this study. 

## 5. Conclusions

The use of ONSs containing HMB along with DC significantly improved body weight, BMI, and nutritional status among community-dwelling older adults at risk of malnutrition in Thailand. These findings underscore the importance of early nutritional intervention in this population. Our study results provide valuable insights to healthcare professionals and policymakers aiming to design targeted nutritional strategies for improving nutritional outcomes and reducing healthcare costs among older adults residing in the community.

## Figures and Tables

**Table 1:** Baseline socio-demographic and characteristics of study participants.

	Overall (*n* = 194)	Control (*n* = 97)	Intervention (*n* = 97)	*p* Value (Between Groups)
Age (years) *	67 (63, 72)	67 (63, 71)	68 (63, 73)	0.5168
Gender, n (%)				0.8485
Male	33 (17.0)	17 (17.5)	16 (16.5)	
Female	161 (83.0)	80 (82.5)	81 (83.5)	
Number of children, n (%)				0.6005
0	107 (55.4)	55 (57.3)	52 (53.6)	
1	27 (14.0)	11 (11.5)	16 (16.5)	
2	31 (16.1)	14 (14.6)	17 (17.5)	
≥3	28 (14.5)	16 (16.7)	12 (12.4)	
Highest level of education, n (%)				0.6830
Primary/Equivalent	59 (30.6)	28 (29.2)	31 (32.0)	
Secondary/Middle/Equivalent	35 (18.1)	19 (19.8)	16 (16.5)	
Diploma/High School/Equivalent	20 (10.4)	12 (12.5)	8 (8.2)	
University and Above	79 (40.9)	37 (38.5)	42 (43.3)	
Main work status over the last 12 months, n (%)				0.5821
Working	18 (9.3)	11 (11.5)	7 (7.2)	
Homemaker/Housewife/Househusband	59 (30.6)	27 (28.1)	32 (33.0)	
Retired	101 (52.3)	49 (51.0)	52 (53.6)	
Unemployed-able to work	15 (7.8)	9 (9.4)	6 (6.2)	
Living area, n (%)				0.8505
Urban area	159 (82.4)	80 (83.3)	79 (81.4)	
Rural area	34 (17.6)	16 (16.7)	18 (18.6)	
Total Charlson Comorbidity Score, n (%)				0.3294
0	182 (94.3)	93 (96.9)	89 (91.8)	
1	9 (4.7)	3 (3.1)	6 (6.2)	
2	1 (0.5)	0 (0.0)	1 (1.0)	
3	1 (0.5)	0 (0.0)	1 (1.0)	
Body weight (kg) ^#^	42.96 ± 0.37	42.96 ± 0.51	42.95 ± 0.53	0.9958
Height (cm) ^#^	153.64 ± 0.52	153.53 ± 0.75	153.76 ± 0.71	0.6824
BMI (kg/m^2^) *	18.4	18.4	18.5	0.9219
	(17.40, 19.20)	(17.45, 19.20)	(17.40, 19.20)	
MUST Score, n (%)				0.8340
1	95 (49.0)	45 (46.4)	50 (51.5)	
2	95 (49.0)	50 (51.4)	45 (46.4)	
3	4 (2.1)	2 (2.1)	2 (2.1)	

^#^ Values are mean ± standard error; * values are median (Q1, Q3); age, gender, MUST risk score, and height were analyzed based on 97 participants from the control group. Other variables were analyzed based on 96 participants from the control group due to availability of the data.

**Table 2:** Dietary intake in the intervention group compared to the control group.

Dietary Intake	Day	Control (n = 97)	Intervention (n = 97)	*p* Value (Between Groups)
Energy (kcal)	Baseline	1171 ± 33	1129 ± 33	0.3724
	30	1207 ± 42	1751 ± 42	<0.0001
	60	1259 ± 40	1795 ± 40	<0.0001
Protein (g)	Baseline	45.27 ± 1.84	44.70 ± 1.84	0.8254
	30	48.06 ± 2.60	70.56 ± 2.62	<0.0001
	60	52.61 ± 2.34	69.75 ± 2.35	<0.0001
Carbohydrate (g)	Baseline	167.8 ± 5.6	154.2 ± 5.6	0.0861
	30	166.0 ± 6.9	240.3 ± 7.0	<0.0001
	60	171.6 ± 6.9	250.7 ± 6.9	<0.0001
Fat (g)	Baseline	35.40 ± 1.77	37.00 ± 1.77	0.5229
	30	40.00 ± 2.58	57.87 ± 2.60	<0.0001
	60	41.37 ± 2.48	58.37 ± 2.50	<0.0001

Values presented are least squares mean ± standard error from the repeated measures ANCOVA (study center, visit, study group, gender, baseline MUST risk, study group*visit, study group*gender, study group*baseline MUST risk) using baseline value as covariate for day 30 and day 60. For baseline comparison between the groups, ANOVA with study center and study group was performed. The sample sizes for some variables are less than the overall stated sample sizes.

## Data Availability

The data presented in this study are available on reasonable request from the corresponding author.

## Supplementary Materials

The following supporting information can be downloaded at: https://www.mdpi.com/article/10.3390/geriatrics9040104/s1, Table S1: Composition of study product per serving.
