# Supplementary material for: Effects of a Specialized Oral Nutritional Supplement with Dietary Counseling on Nutritional Outcomes in Community-Dwelling Older Adults at Risk of Malnutrition: A Randomized Controlled Trial
Source: Geriatrics (Basel). 2024 Aug 17;9(4):104. doi: 10.3390/geriatrics9040104 (PMC11353685; doi:10.3390/geriatrics9040104)
Supplement: Supplementary file 1 [file geriatrics-09-00104-s001.zip › geriatrics-3084990-supplementary.pdf]

Supplementary Materials S1: Table S1: Composition of study product per serving.

	Unit	Oral Nutritional Supplement (Intervention Group)
Energy	kcal	270
Protein	g	11
Fat	g	9
Carbohydrate	g	34.5
CaHMB	g	0.74
Vitamin D_3	mcg	7.7
Vitamin E	mg α-TE	4.8
Vitamin C	mg	44
Calcium	mg	275
Zinc	mg	4.0
Selenium	mcg	20
